# Theoretical investigation of the MXene precursors Mo_x_V_4-x_AlC_3_ (0 ≤ x ≤ 4)

**DOI:** 10.1038/s41598-023-30443-z

**Published:** 2023-02-25

**Authors:** Ma. Guadalupe Moreno-Armenta, J. Guerrero-Sánchez, S. J. Gutiérrez-Ojeda, H. N. Fernández-Escamilla, D. M. Hoat, R. Ponce-Pérez

**Affiliations:** 1 Centro de Nanociencias y Nanotecnología, Universidad Nacional Autónoma de México, Km. 107, Apdo. Postal 14. Carretera Tijuana-Ensenada, Ensenada, Baja California México; 2 Facultad de Ciencias Físico Matemáticas, Universidad Autónoma de Nuevo León, 66451 San Nicolas de los Garza, Nuevo León México; 3 Institute of Theoretical and Applied Research, Duy Tan University, Ha Noi, 100000 Vietnam; 4 Faculty of Natural Sciences, Duy Tan University, Da Nang, 550000 Vietnam

**Keywords:** Condensed-matter physics, Nanoscale materials, Theory and computation

## Abstract

By first-principles total-energy calculations, we investigated the thermodynamic stability of the MAX solid solution Mo_x_V_4-x_AlC_3_ in the 0 ≤ x ≤ 4 range. Results evidence that lattice parameter *a* increases as a function of Mo content, while the *c* parameter reaches its maximum expansion at x = 2.5. After that, a contraction is noticed. Mo occupies V_I_ sites randomly until the out-of-plane ordered Mo_2_V_2_AlC_3_ alloy is formed. We employed the Defect Formation Energy (DFE) formalism to evaluate the thermodynamic stability of the alloys. Calculations show five stable compounds. At V-rich conditions and from Mo-rich to Mo-moderated conditions, the pristine V_4_AlC_3_ MAX is stable. In the region of V-poor conditions, from Mo-rich to Mo-moderated growth conditions, the solid solutions with x = 0.5, 1, and 1.5 and the o-MAX Mo_2_V_2_AlC_3_ are thermodynamically stable. The line profiles of the Electron Localization Function and Bader charge analysis show that the V-C interaction is mainly ionic, while the Mo-C is covalent. Also, the exfoliation energy to obtain a MXene layer is ~ 0.4 eV/Å^2^. DFE also shows that MXenes exfoliated from the MAX phase with the same Mo content and atomic arrangement are thermodynamically stable. Our results get a deeper atomic scale understanding of the previously reported experimental evidence.

## Introduction

The MAX compounds are a family of hexagonal layered early-transition metal carbides, nitrides, or carbonitrides with general formula M_n+1_AX_n_^1^, where M is an early-transition metal, A is typically an atom of the group 13 to 16, and X is carbon/nitrogen, with n = 1, 2, and 3. Depending on the number of M layers, the shorthand notation can be 211, 312, 413, with n = 1, 2, or 3, respectively. Also, in the last years, the higher order MAX compounds (n > 3) have been successfully synthesized, such as Ti_7_SnC_6_^2^, Ta_6_AlC_5_^3^, (Ti_0.5_Nb_0.5_)_5_AlC_4_^4^, (V_0.5_Cr_0.5_)_5_Al_2_C_3_^5^ and Mo_4_VAlC_4_^6^. The MAX compounds exhibit a combination of metallic and ceramic properties. They have high electrical and thermal conductivity and tribological properties. They are machinable, and present high damage tolerance and thermal-shock resistance. Also, their strength remains at high temperatures^7–13^. Thanks to their properties, the MAX compounds can be employed in catalysis^14,15^, in nuclear reactors^16,17^, and, as high-temperature protective coatings^18^. The MAX compounds with Al as an A element, such as Ti_2_AlC, Cr_2_AlC, and Ti_3_AlC_2,_ are good candidates for high-temperature applications since they provide high oxidation resistance and form a protective alumina layer (Al_2_O_3_)^19–21^. Also, MAX compounds are the precursors of the two-dimensional MXenes discovered in 2011^22,23^.

The Mxenes are exfoliated from the precursor MAX phase by selectively etching the A element. They possess excellent properties making them suitable materials for many applications. In the past year, the first evidence of ferroelectricity and multiferroicity in Mxenes at room temperature was obtained^24^. For hydrogen production through water splitting, the bifunctional catalysts Mo_2_TiC_2_ and Mo_2_Ti_2_C_3_ have demonstrated outstanding performance in alkaline media^25^. The Ti_4_C_3_ Mxene is an electrode for carbon-supported memory applications based on Ti_4_C_3_/graphene oxide/Ti_4_C_3_ frameworks^26^, with excellent retention and endurance characteristics. About energy storage devices, the double-ordered alloy Ti_2_Ta_2_C_3_ is an efficient electrode in Li-ion batteries, better than the pristine Ti_4_C_3_ Mxene^27,28^. Also, some reports suggest improved storage properties of the Mxenes by combining them with ZrO_2_, MnO_2_, and metallic dopants, such as Ni. In some cases, the modified materials can double the capacitance compared to pristine materials. Also, their capacitance is larger than 81% up to 10,000 cycles^29–31^.

Since the discovery of the Cr_2_TiAlC_2_ ordered structure by Liu et al.^32^ in 2014 -Ti is sandwiched between two chromium carbide layers in a 312 structure-, a new subclass of MAX compounds with formula (M, M’)_n+1_AlX_n_ has attracted attention thanks to their chemical versatility. In the last few years, Cr- and Mo-based MAX have been synthesized with small amounts of Fe or Mn to improve their magnetic properties^33,34^.

In the case of the 211 MAX compounds, the alloys are in-plane (i-MAX) ordered, where the M element forms hexagons and the M’ element is at the center. An example of this is the (Mo_2/3_Sc_1/3_)_2_AlC MAX alloy synthesized in 2017^35,36^. In the last years, the (V_2/3_Zr_1/3_)_2_AlC, (Cr_2/3_Sc_1/3_)_2_AlC, (Cr_2/3_Sc_1/3_)_2_GaC, (W_2/3_Sc_1/3_)_2_AlC, (Mn_2/3_Sc_1/3_)_2_GaC i-MAX compounds have been successfully growth^1^. Furthermore, in 312 and 413 MAX phases, the out-of-plane ordering is the most common (o-MAX). M’ is placed at the center of the carbide or nitride layer, forming an octahedral coordination with the C (N) atoms, while the M element appears in most exposed layers. In the last years, the o-MAX Mo_2_TiAlC_2_ and Mo_2_TiAlC_2_ have been synthesized. In both cases, Mo occupies the most exposed carbide layers, and Ti is at the inner layers^37,38^. This behavior has been investigated by theoretical calculations, demonstrating that Mo avoids face-centered cubic (FCC) stackings when coordinated with C since there is energy loss when adopting this configuration^39^.

Although the out-of-plane arrangement is the most common in the 312 and 413 double transition metals (DTM) MAX, the formation of solid solutions is another possibility. Griseri et al.^40^ synthesized Ta-based 413 MAX solid solutions with Hf and Nb to form (Ta_1-x_Hf_x_)_4_AlC_3_ and (Ta_1-x_Nb_x_)_4_AlC_3_ with x = 0.05, 0.1, 0.15, 0.2 and 0.25, they included Hf in the structure to form ultra-high temperature ceramics, as previously predicted by DFT calculations^41,42^. Qu and coworkers^43^, by Spark Plasma Sintering (SPS), obtained the (Ti_1-x_Zr_x_)_3_SiC_2_ solid solution, x ranging up to 0.17. They found an anisotropic expansion of the cell parameter as Zr content increases. Recently, Pinto et al.^44^ synthesized for the first time the solid solution Mo_x_V_4-x_AlC_3_ with x = 1, 1.5, 2, and 2.7. For concentrations larger than x = 2.7, the compound obtained was not identified as the MAX phase. The XRD pattern shows lattice parameters' linear expansion -as a function of Mo content- for the entire range of Mo content. However, in this report, a detailed characterization of the atomic arrangement is still needed. After that, MXenes were obtained by selective topochemical etching. Finally, the MXene layers were applied in energy storage devices, obtaining good results. Motivated by the findings of Pinto et al.^44^, in this work, we investigated the atomic arrangements of the Mo_x_V_4-x_AlC_3_ solid solutions considering the entire range of concentrations (from x = 0 to x = 2). Our results show that Mo occupies V sites in a semi-ordered arrangement until reaching the Mo_2_V_2_AlC_3_ o-MAX. The manuscript is organized as follows: section II is for the computational methodology, the results are presented in section III, and conclusions are made in section IV.

## Method

Through a systematic computational assessment, we investigated the binary MAX alloy Mo_x_V_4-x_AlC_3_ with 0 ≤ x ≤ 4. Calculations were performed within the periodic Density Functional Theory (DFT) as implemented in the Vienna Ab initio Simulation Package (VASP)^45–48^. Exchange–correlation energies are approximated using the generalized gradient approximation (GGA) and the Perdew-Burke-Ernzerhof (PBE) parametrization^49^. Electronic states are treated as a plane-waves with an energy cutoff of 460 eV. The frozen core approximation has been employed through the projector-augmented-wave (PAW) method^50,51^. Van der Waals forces were considered in the calculations employing the DFT-D3 method of Grimme^52,53^. Geometry optimization is achieved when all force components are less than 0.01 eV/Å, and the energy differences less than 1 × 10^–4^ eV. A 3 × 3 × 1 supercell has been considered to investigate the different V: Mo stoichiometries. To sample the Brillouin zone, we used the equally spaced k-points scheme of Monkhorst–Pack^54^, with a k-points grid of 3 × 3 × 2. The Climbing Image Nudged Elastic Band (CI-NEB) method^55,56^ is employed to calculate the exfoliation energy, considering seven intermediate images from the initial to final stages.

### Defect formation energy formalism

We are interested in evaluating the thermodynamic stability of the binary MAX Mo_x_V_4-x_AlC_3_ compound, with 0 ≤ x ≤ 4. The total energy criterion is not enough since each V:Mo relationship has different number of atoms and chemical species. Therefore, we must employ the Defect Formation Energy (DFE) formalism^57–60^ which is independent of the number of atoms in the system and only depends on the chemical potentials of the constituent species. Thermodynamic equilibrium is required to apply the formalism, this implies:

1 $$4{\mu }_{V}^{bulk}+{\mu }_{Al}^{bulk}+3{\mu }_{C}^{bulk}-{\Delta H}_{{V}_{4}Al{C}_{3}}^{f}={\mu }_{{V}_{4}Al{C}_{3}}=4{\mu }_{V}+{\mu }_{Al}=3{\mu }_{C},$$

where, μ_i_ is the chemical potential of the ith species and ${\Delta H}_{{V}_{4}Al{C}_{3}}^{f}$ is the formation enthalpy. The DFE formalism can be write as follows:

2 $${E}^{DFE}=\frac{1}{{V}_{UC}}\left[{E}^{slab}-{n}_{Al}{\mu }_{{V}_{4}Al{C}_{3}}-{\mu }_{V}\left({n}_{V}-4{n}_{Al}\right)-{\mu }_{C}\left({n}_{C}-3{n}_{Al}\right)-{\mu }_{Mo}{n}_{Mo}\right],$$

where, ${E}^{slab}$ is the total energy of the system at hand, V_UC_ is the volume of the unit cell, and n_i_ is the number of atoms of the ith species. V thermodynamic limit is defined by the heat of formation of the V_4_AlC_3_ MAX with:

3 $${\mu }_{V}^{bulk}-\frac{1}{4}{\Delta H}_{{V}_{4}Al{C}_{3}}^{f}\le {\Delta \mu }_{V}\le {\mu }_{V}^{bulk},$$

also, the variation of the Mo chemical potential is limited by:

4 $${\mu }_{Mo}^{bulk}-{\mu }_{Mo}^{cohesive}\le {\Delta \mu }_{Mo}\le {\mu }_{Mo}^{bulk},$$

with ${\mu }_{Mo}^{cohesive}$ as the cohesive energy of Mo.

## Results

**Figure 1:**
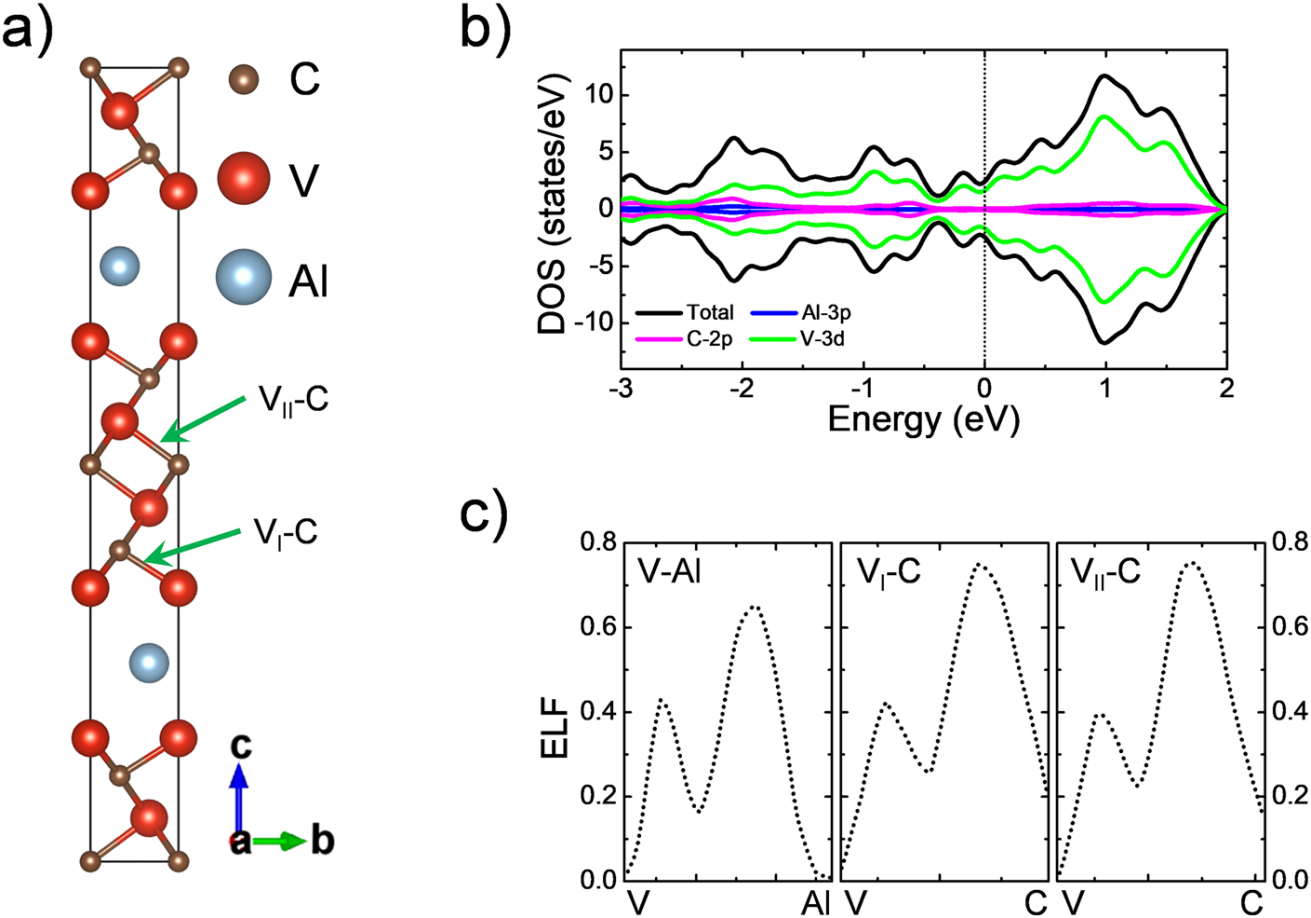
(**a**) atomistic model of the V_4_AlC_3_ MAX compound, (**b**) Total and projected density of states, and (**c**) the line profiles of electron localization function.

To investigate the thermodynamic stability of the Mo_x_V_4-x_AlC_3_ solid solution, we choose the previously reported α-V_4_AlC_3_ MAX compound^61^ as the basis for the Mo substitutions. α-V_4_AlC_3_ (from now on V_4_AlC_3_) has space group P6_3_/mmc with lattice parameter a = 2.92 Å and c = 22.75 Å. V atoms occupy 4f. (1/3, 2/3, 0.05441) and 4e (0, 0, 0.15481) Wyckoff sites, while C the 2a (0, 0, 0) and 4f. (2/3, 1/3, 0.10802) sites. Also, Al is placed in the 2c (1/3, 2/3, 1/4) Wyckoff site. Calculated MAX structure exhibits cell parameters a = 2.90 Å and c = 22.57 Å, in good agreement with experimental reports^61^. Figure 1a shows the V_4_AlC_3_ atomic model and its respective Density of States (DOS) (Fig. 1b), where the energy reference is the Fermi level. The structure depicts metallic characteristics. The V-3d orbitals have the most important contributions to the DOS. Each C atom is six-fold coordinated to V. Two kinds of V atoms can be distinguished: V_I_ corresponds to the most exposed V, those that interact with Al and C atoms, and the V_II_ that only interact with C atoms (see Fig. 1). The V_II_-C bond is 2.08 Å, while the bond distance between the C and the V_I_ is ~ 1.98 Å. The Electron Localized Function (ELF) is employed to investigate the nature of the bonds present in the MAX phase, the isosurface with a value of 0.6 a.u. is shown in the Fig. S1 of the Supplementary Information (SI). The line profile across the different bonds present in the MAX is displayed in Fig. 1c. The V_II_-C bonds are mainly ionic. Also, the V_I_-C depicts a similar behavior; however, a large electron population appears in the middle of the bond, suggesting a stronger interaction, which explains the bond distances. Also, the ELF line profiles show that the V_I_-Al bond is the weakest, with a length of 2.71 Å. It has been reported that in the MAX compound, a weak interaction is accompanied by a strong interaction^43,62^. In our case, V_I_ strongly/weakly interacts with C/Al, so delamination of the structure is possible.

### Formation of the V_x-1_Mo_x_AlC_3_ alloy

Previously, it has been reported the successful synthesis of the Mo_x_V_4-x_AlC_3_ MAX solid solution with different stoichiometries (0 ≤ x ≤ 2.7)^44^. By first-principles calculations and first-principles thermodynamics, we evaluated the stability of different alloys for the entire range 0 ≤ x ≤ 4. Recall that a 3 × 3 × 1 periodicity is enough to model the right stoichiometry for each case. Different atomic arrangements were considered, in which Mo is randomly placed in V_I_ and V_II_ sites. Also, semi-ordered arrangements were accounted for to evaluate their stability. Table 1 summarizes the relative energies of the first four stable models considered in this work for each of the different stoichiometries. Model I is the reference and the most stable configuration in all cases. See the atomistic models of the structures in Figs. S2 and S3. For the range 0 ≤ x ≤ 2, we notice that Mo substitutions have a semi-disordered arrangement, i.e., Mo randomly substitutes V_I_ sites (see model I in Fig. S2) until they form the Mo_2_V_2_AlC_3_ compound. In the second place of stability are the models where Mo occupies V_I_ sites of a single monolayer. The less stable configurations are those in which Mo randomly substitutes V_I_ and V_II_ sites. Note that for the Mo_2_V_2_AlC_3_ compound, the MAX phase adopts an out-of-plane chemically ordered structure, like Ti_2_Ta_2_C_3_ or Mo_2_Ti_2_C_3_^28,37^.

**Table 1:** Relative energy (in eV) for the first four most stable V_x-1_Mo_x_AlC_3_ MAX alloy models.

MAX alloy	Model (eV)			
	I	II	III	IV
Mo_0.5_V_3.5_AlC_3_	0.00	0.47	1.41	3.35
MoV_3_AlC_3_	0.00	1.46	2.33	3.59
Mo_1.5_V_2.5_AlC_3_	0.00	0.35	4.07	5.38
Mo_2_V_2_AlC_3_	0.00	5.12	6.93	7.43
Mo_2.5_V_1.5_AlC_3_	0.00	0.29	5.10	5.63
Mo_3_VAlC_3_	0.00	0.73	4.26	9.47
Mo_3.5_V_0.5_AlC_3_	0.00	0.33	3.44	3.85

For Mo concentrations larger than x = 2, the most stable model in each case is formed by the full substitution of the V_I_ sites and a random arrangement of Mo in the V_II_ sites, see models I in Fig. S3. The less stable models are formed by the random deposition of Mo in V_I_ and V_II_ sites or by alternating Mo and V layers along the c direction.

**Figure 2:**
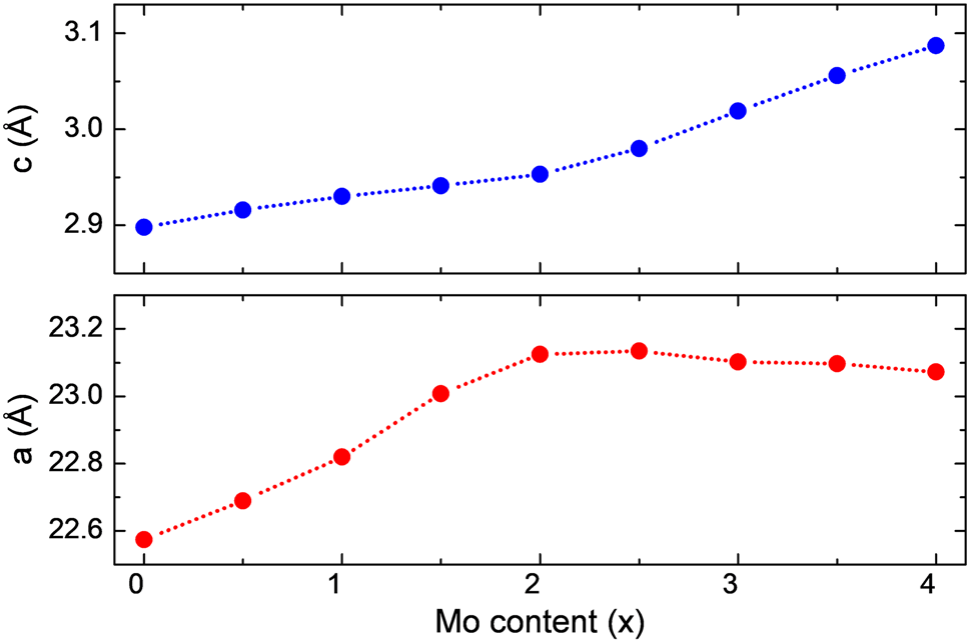
Cell parameters of the Mo_x_V_4-x_AlC_3_ MAX alloy as a function of the Mo content. The upper and lower panels are for cell parameters a and c, respectively.

We also investigated the lattice parameter evolution vs. Mo content (see Fig. 2). The upper and lower panels correspond to a and c lattice parameters. The a parameter expands from 2.90 Å to 3.09 Å (6.5%), from x = 0 to x = 4, which fulfills Vegard’s law. On the other hand, c parameter achieves its maximum expansion (2.49%, 23.14 Å) at x = 2.5. After that, the cell parameter slightly contracts, reaching 23.07 Å for x = 4, which violates Vegard’s law. This fact can be attributed to the large electronegativity difference between Mo and V and the presence of covalent bonds, as previously reported for different compounds^63,64^. According to the calculations, the Mo_x_V_4-x_AlC_3_ MAX alloy follows Vegard’s law until x = 2.5, demonstrating that our theoretical results agree with the experimental findings of Pinto et al.^44^.

The changes in the different bonds present in the alloy are investigated. The results are listed in Table 2. Notice that when increasing the Mo content in the structure, the bonds suffer an expansion, which is an expected result since Mo has a large atomic radius. The V_I_-C is expanded from 1.98 Å for the V_4_AlC_3_ to 2.00 Å for the Mo_1.5_V_2.5_AlC_3_ MAX alloy. About V_II_-C at Mo concentrations lower than x = 2, the bond distance remains practically unaltered since Mo only occupies V_I_ sites. However, for higher concentrations (x > 2), the bond distance increases considerably due to the occupation of the V_II_ sites. Moreover, Mo occupying V_I_ sites (Mo_I_) provides large bond distances compared to V_I_-C. At concentrations higher than x = 2, Mo occupies V_II_ sites (Mo_II_). Mo_II_ generates larger bond distances than V_II_, which rises as the Mo content goes from 2.13 Å in Mo_2.5_V_1.5_AlC_3_ to 2.19 in Mo_4_AlC_3_.

**Table 2:** Calculated bond distances present in the Mo_x_V_4-x_AlC_3_ MAX alloy for the entire range 0 ≤ x ≤ 4.

MAX alloy	Bond length (Å)				
	V_I_-C	V_II_-C	Al interlayer distance	Mo_I_-C	Mo_II_-C
V_4_AlC_3_	1.98	2.08	2.14	–	–
Mo_0.5_V_3.5_AlC_3_	1.99	2.08	2.14	2.06	–
MoV_3_AlC_3_	1.99	2.09	2.19	2.06	–
Mo_1.5_V_2.5_AlC_3_	2.00	2.09	2.21	2.07	–
Mo_2_V_2_AlC_3_	–	2.09	2.21	2.07	–
Mo_2.5_V_1.5_AlC_3_	–	2.11	2.19	2.08	2.13
Mo_3_VAlC_3_	–	2.14	2.19	2.07	2.15
Mo_0.5_V_3.5_AlC_3_	–	2.14	2.19	2.07	2.19
Mo_4_AlC_3_	–	–	2.20	2.05	2.20

### Thermodynamic stability

**Figure 3:**
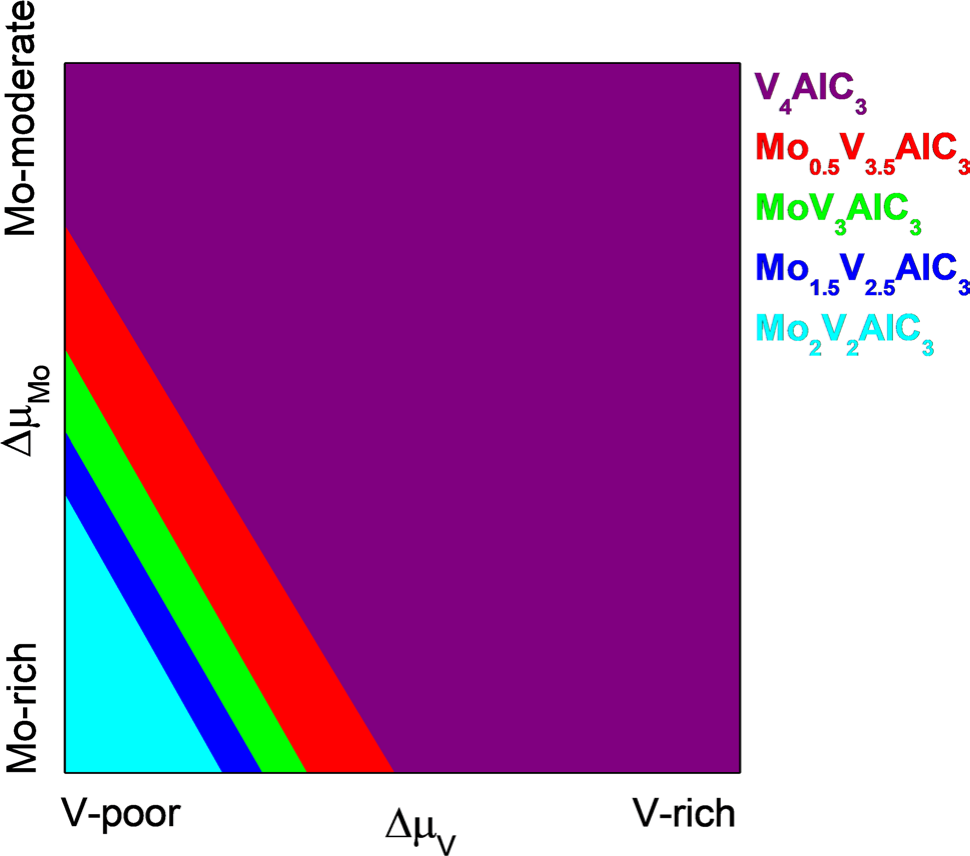
Corresponding phase diagram of the MAX Mo_x_V_4-x_AlC_3_ alloy.

**Figure 4:**
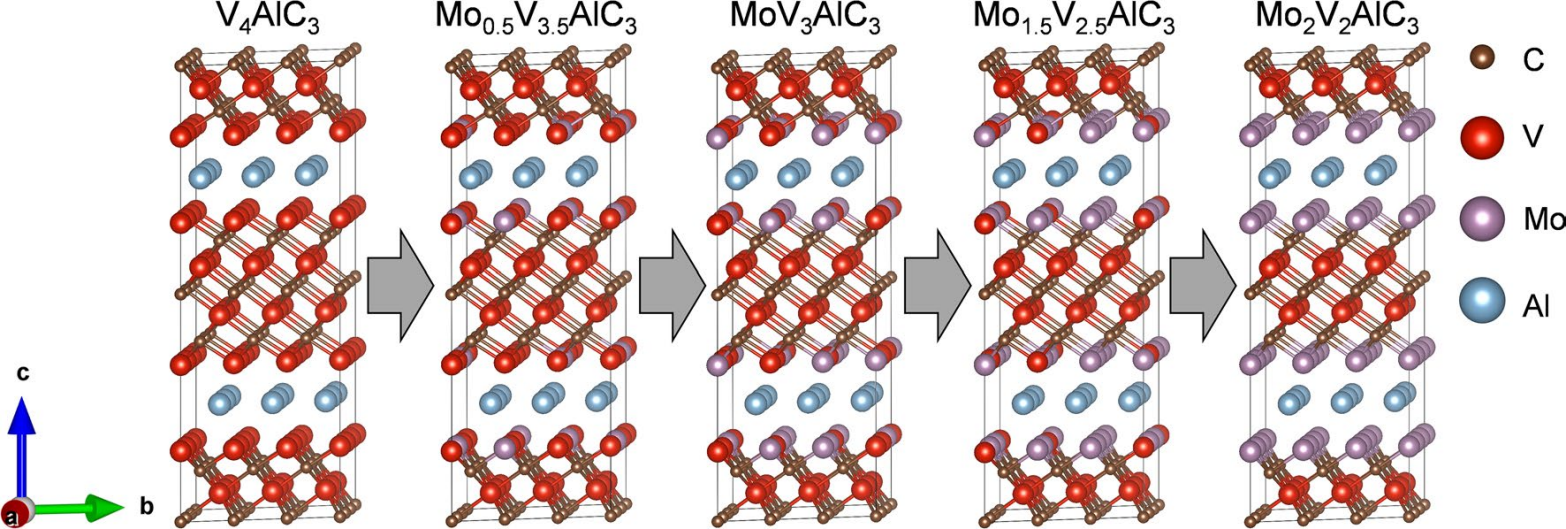
Atomistic models for the five stable MAX alloys according to the DFE formalism. We show the models as the Mo content increases from left to right.

We employed the DFE formalism described in the methodology section to investigate the thermodynamic stability of the different alloys. We only considered the most stable configuration for each Mo content according to Table 1. We vary the chemical potential from V-rich to V-poor conditions and from Mo-rich to Mo-moderate conditions to evaluate the thermodynamic stability of the MAX alloys. We do not consider Mo-poor conditions since the pristine MAX phase is the only stable in that chemical potential region. Figure S4 shows the 3D plot, where each plane represents a different alloy. According to our formalism, the most stable models depict the lowest energy values. Although the 3D representation helps distinguish the stable models, it is difficult to differentiate the chemical potential regions where the models are stable. We project the 3D plot into a 2D phase diagram with well-defined growth limits to solve this problem. Figure 3 displays the phase diagram for the Mo_x_V_4-x_AlC_3_ MAX solid solution. At V-rich and Mo-moderate conditions, the pristine V_4_AlC_3_ compound is the most stable model (purple surface). Notice that the Mo doping is carried out in the region from V-moderate to V-poor conditions and from Mo-moderate to Mo-rich conditions. Our calculations show four stable and different Mo_x_V_4-x_AlC_3_ (with x = 0.5,1, 1.5, and 2) alloys. Also, it is noticed that as the μ_V_ trend to V-poor and the μ_Mo_ moves to Mo-rich conditions, the Mo content increases until reaching the double-ordered alloy V_2_Mo_2_AlC_3_. The atomistic models of the most stable models are shown in Fig. 4. Our DFE analysis agrees with the experimental results reported by Pinto et al.^44^. However, they successfully obtained the Mo_2.7_V_1.3_AlC_3_ alloy disagrees with our findings. A possible explanation is that the alloy mentioned above can be obtained due to border effects -not considered in our study- in which V_II_ sites are not in an FCC stacking, generating an energy gain when Mo occupies those sites.

### Charge distribution

In this section, we perform a Bader charge analysis in combination with ELF line profiles to investigate the nature of the bonding interactions in the stable systems. Note from the line profiles that ELF tends to zero as it reaches the atoms’ core due to using pseudopotentials. Bader analysis of the pristine V_4_AlC_3_ MAX shows that the V_II_ atoms donate 0.25e to each of its six C neighbors. V_I_ atoms donate 0.28e to their neighboring C atoms; this change evidence a stronger interaction in V_I_-C than in V_II_-C. This effect is seen in the ELF line profile (see Fig. 1c), where it is noticed that both bonds exhibit a mainly ionic behavior. Also, it is notorious for a major population of electrons along the V_I_-C bond. Moreover, the Al atoms accept 0.17e from the V nearest neighbor.

In the case of the stable Mo_x_V_4-x_AlC_3_ alloys, where Mo substitutes the V_I_ atoms, we notice that Mo donates 0.23e to each of its bonds formed with C. This behavior supposes that Mo donates fewer electrons in comparison with V. ELF line profiles explain this effect showing a bond nature change from ionic to covalent, see Fig. S5. Despite this, the V_II_-C interactions do not change. V_II_ remains donating 0.25e to each of its six C neighbors (Fig. S5). However, unlike pristine V_4_AlC_3_, Al donates 0.55e to neighboring Mo atoms in the case of the alloys.

### Density of states

In this subsection, we analyzed the DOS of the most stable alloys. The corresponding DOS for Mo_x_V_4-x_AlC_3_ with x = 0.5, 1, 1.5, and 2 are depicted in Fig. S6, SI. In all cases, the energy reference is the Fermi level. Positive and negative values along the DOS axis corresponds with spin up and down, respectively. As in the pristine MAX (see Fig. 1), the alloys exhibit a metallic behavior with non-magnetic characteristics. The main contribution to the DOS around the Fermi level comes from the V-3d orbitals, followed by the Mo-4d orbitals, which increase their contribution to the DOS as Mo content increases.

### Exfoliation energy and MXene formation

**Figure 5:**
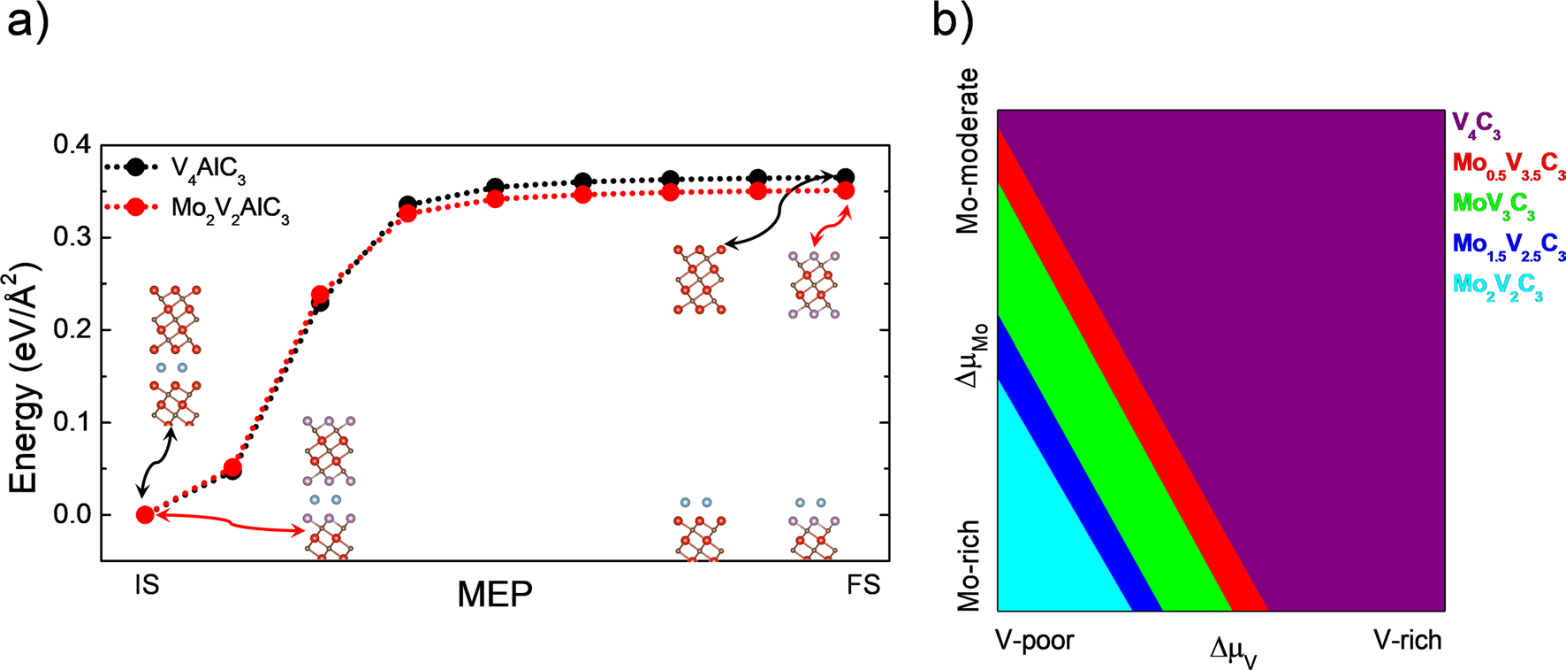
(**a**) minimum energy pathway for the exfoliation of a monolayer of V_4_C_3_ (black points) and Mo_2_V_2_C_3_ (red points) MXenes, (**b**) phase diagram for the thermodynamic stability of the different Mo_x_V_4-x_C_3_ MXenes.

Once we studied the stability and bond nature of the Mo_x_V_4-x_AlC_3_ alloys, we focused on investigating the exfoliation energy of the so-called MXenes. As a first approximation, we do not consider the presence of functional groups on the surface to isolate the Mo content effect in the exfoliation process. We only consider the extreme cases, x = 0 and x = 2. NEB calculations have been performed to investigate the minimum energy pathway (MEP), see Fig. 5a. The energy reference, Initial State (IS), is the V-terminated (Mo-terminated) (0001) surface, and the Final State (FS) occurs when the MXene is far enough to interact with the surface. The MXene exfoliation is an endothermic process; for the V_4_AlC_3_ is 0.37 eV/Å^2^, while for the ordered alloy, Mo_2_V_2_AlC_3_, the energy is 0.35 eV/Å^2^. Results demonstrate that Mo reduces the exfoliation energy, an effect explained through Bader charge analysis and ELF in the previous subsection.

**Figure 6:**
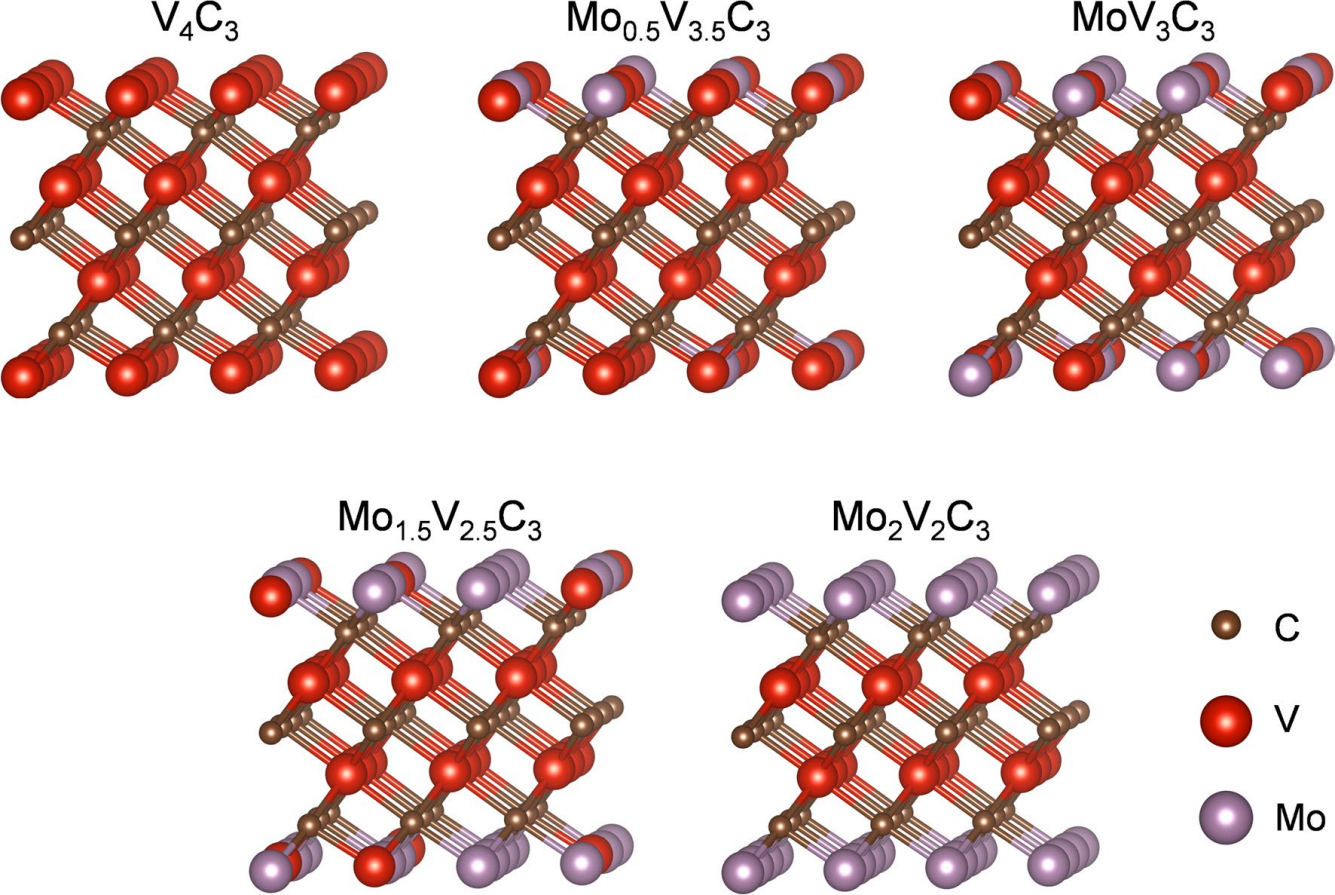
Atomic models of the stable MXenes according to Fig. 5b.

We also investigated the thermodynamic stability of the MXenes in a similar way to the MAX compounds. Their corresponding phase diagram is displayed in Fig. 5b. As we can see, the alloys are stable at V-poor and Mo-rich conditions, while the pristine V_4_C_3_ MXene is stable at V-rich conditions in the entire region from Mo-rich to Mo-moderate conditions. Similarly, to the MAX case, in the MXenes, the Mo content increases as reaching V-poor and Mo-rich conditions until obtaining the double-ordered Mo_2_V_2_C_3_ MXene alloy. The atomistic models of the stable MXenes are depicted in Fig. 6.

## Conclusions

Through a systematic ab-initio assessment, we investigated the thermodynamic stability of the MXene precursor Mo_x_V_4-x_AlC_3_ in the 0 ≤ x ≤ 4 range. Mo incorporation generates an increase in a, while c expands until x = 2.5. Above such concentration, a slight contraction in c appears. Mo incorporates in a random, however, it achieves an order in the out-of-plane Mo_2_V_2_AlC_3_. From all the considered concentrations, we found five thermodynamically stable compounds (V_4_AlC_3_, Mo_0.5_V_3.5_AlC_3_, MoV_3_AlC_3_, Mo_1.5_V_2.5_AlC_3_, and Mo_2_V_2_AlC_3_) at different growth conditions. Electron Localization Function shows that V-C bonds are ionic while Mo-C covalent. Finally, we evaluated the exfoliation energy in the limiting stable cases (V_4_AlC_3_ and Mo_2_V_2_AlC_3_), since the changes in the bonding type induced by the Mo incorporation, the exfoliation of the Mo_2_V_2_AlC_3_ is more favorable. Finally, our results evidence that five MXenes can be obtained from their counterpart MAX phase alloys.

## Supplementary Information

Supplementary Information.

## Data Availability

The datasets used and/or analyzed during the current study are available from the corresponding author on reasonable request.
