# Supplementary material for: Theoretical investigation of the MXene precursors MoxV4-xAlC3 (0 ≤ x ≤ 4)
Source: Sci Rep. 2023 Feb 25;13:3271. doi: 10.1038/s41598-023-30443-z (PMC9968326; doi:10.1038/s41598-023-30443-z)
Supplement: Supplementary file 1 — Supplementary Information. [file 41598_2023_30443_MOESM1_ESM.docx]

**Supplementary information of the manuscript ‘Theoretical investigation of the MXene precursors Mo_x_V_4-x_AlC_3_ (0≤x≤4)’**

Ma. Guadalupe Moreno-Armenta ^a^, J. Guerrero-Sanchez ^a^, S. J. Gutierrez-Ojeda ^a^, H. N. Fernandez-Escamilla ^b^, D. M. Hoat ^c, d^, R. Ponce-Perez^a^

*a) Centro de Nanociencias y Nanotecnología, Universidad Nacional Autónoma de México, Apartado Postal 14, Código Postal, Ensenada, Baja California 22800, México*

*b) Facultad de Ciencias Físico Matemáticas, Universidad Autónoma de Nuevo Leon, San Nicolas de los Garza, San Nicolas de los Garza, Nuevo León, 66451, México.*

*c) Institute of Theoretical and Applied Research, Duy Tan University, Ha Noi, 100000, Vietnam*

*d) Faculty of Natural Sciences, Duy Tan University, Da Nang, 550000, Vietnam*

* Corresponding email: [moreno@ens.cnyn.unam.mx](mailto:moreno@ens.cnyn.unam.mx) (MGMA), [rponce@ens.cnyn.unam.mx](mailto:rponce@ens.cnyn.unam.mx) (RPP)

ELF isosurface with a value of 0.6 a.u. for the V_4_AlC_3_ MAX compound is shown in **figure S1**, an ionic interaction is observed between V and C atoms, while the Al monolayer posessess a metallic behavior.


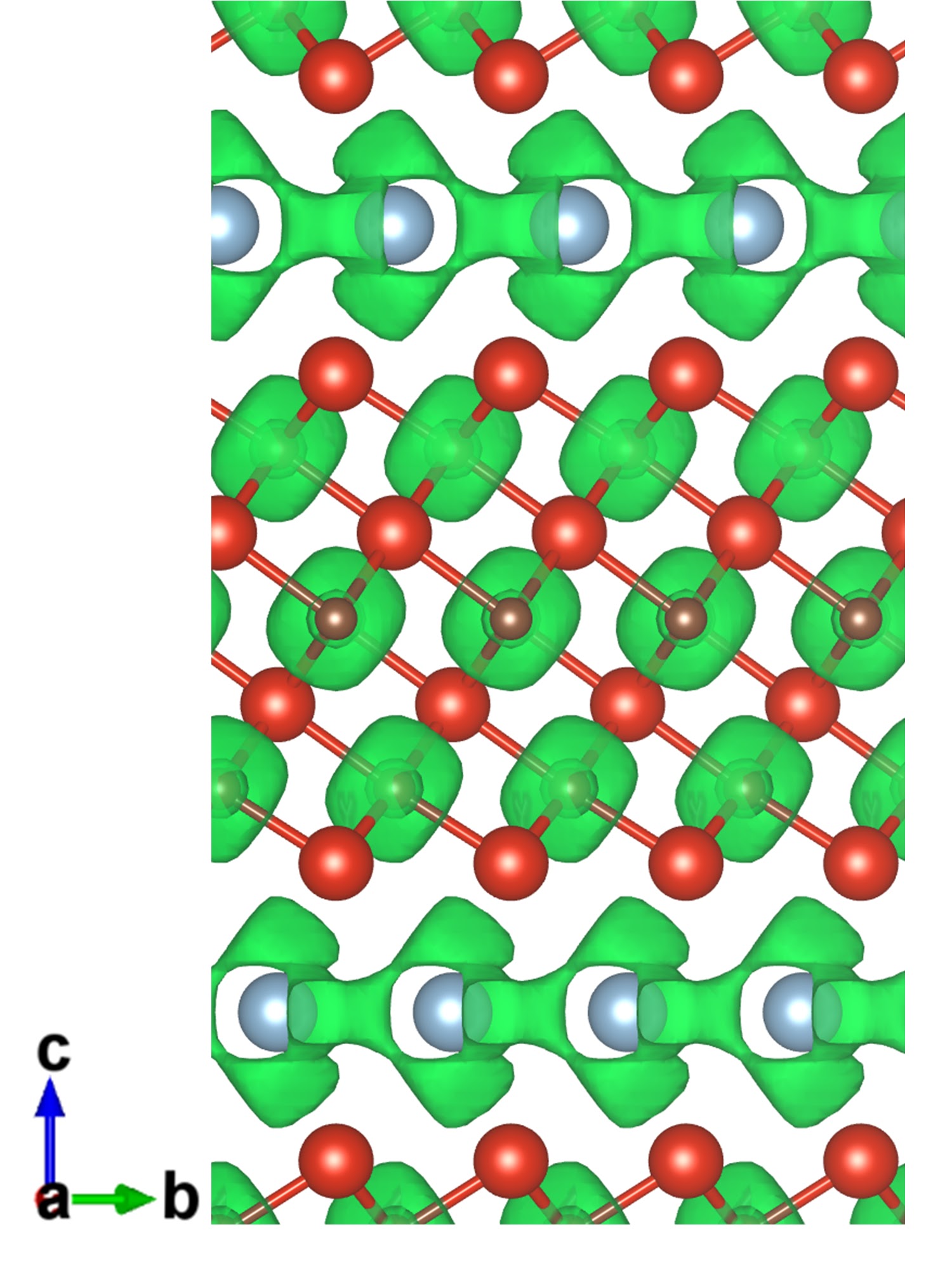


**Figure S1**. ELF isosurface, isovalue 0.6 a.u., for the V_4_AlC_3_ MAX compound.

**Figure S2** shows the atomistic models for the four most stable configurations of the Mo_x_V_4-x_AlC_3_ alloy in the range from x= 0.5 to x= 2. **Figure S3** displays the most stable configurations from x= 2.5 to x= 3.5. In both cases, the structures are ordered from left to right in order of stability. Mo occupies the V_I_ sites until it forms the out-of-plane Mo_2_V_2_AlC_3_ MAX alloy (x=2). After that, Mo settles in V_II_ sites, where it again occupies sites in random order.


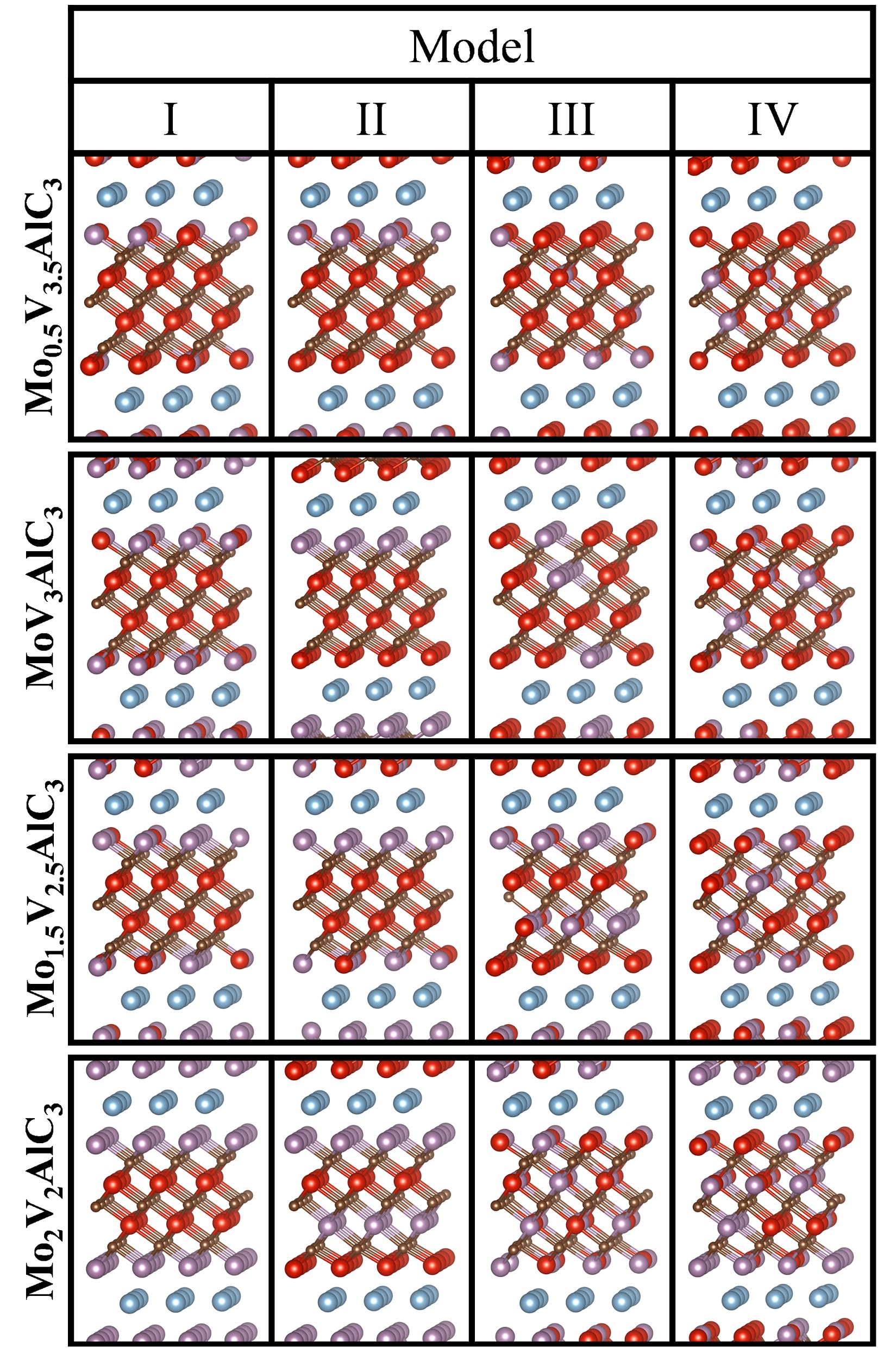


**Figure S2**. The first four stable models considered in the calculations for the Mo_x_V_4-x_AlC_3_ in the range 0.5≤x≤2. In all cases the stability order is from left to right.


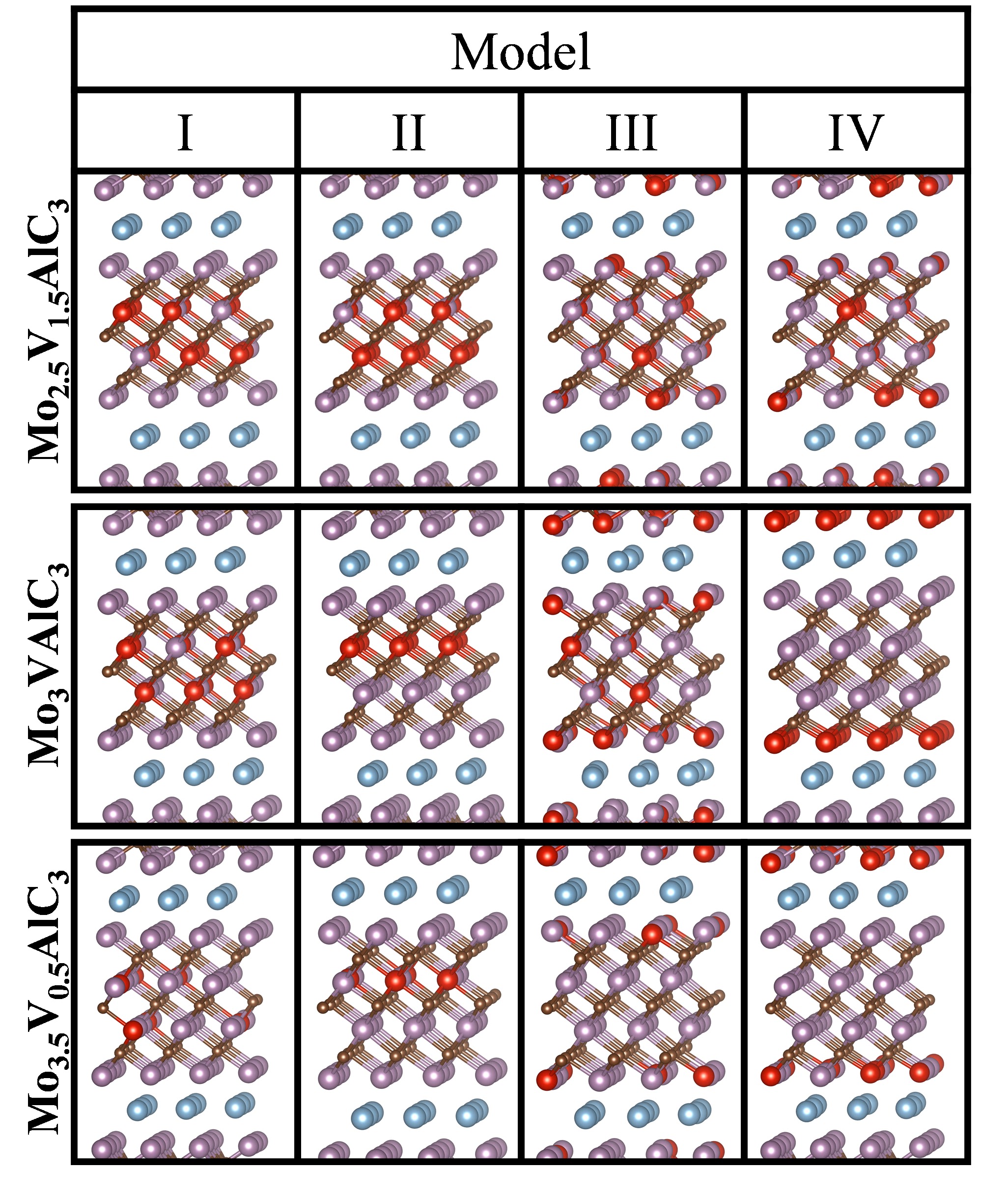


**Figure S3**. The first four stable models considered in the calculations for the Mo_x_V_4-x_AlC_3_ in the range 2.5≤x≤3.5. In all cases the stability order is from left to right.

The three-dimensional representation of the DFE for the Mo_x_V_4-x_AlC_3_ alloy is shown in **figure S4**, where each plane represents a different alloy. According to the DFE formalism, the most stable structures provide the lowest energy values, therefore, we focus in the bottom part of the graph, the colors purple, red, green, blue, cyan, magenta, dark yellow, navy and black corresponds to V_4_AlC_3_, Mo_0.5_V_3.5_AlC_3_, MoV_3_AlC_3_, Mo_1.5_V_2.5_AlC_3_, Mo_2_V_2_AlC_3_, Mo_2.5_V_1.5_AlC_3_, Mo_3_VAlC_3_, Mo_3.5_V_0.5_AlC_3_, Mo_4_AlC_3_, respectively.


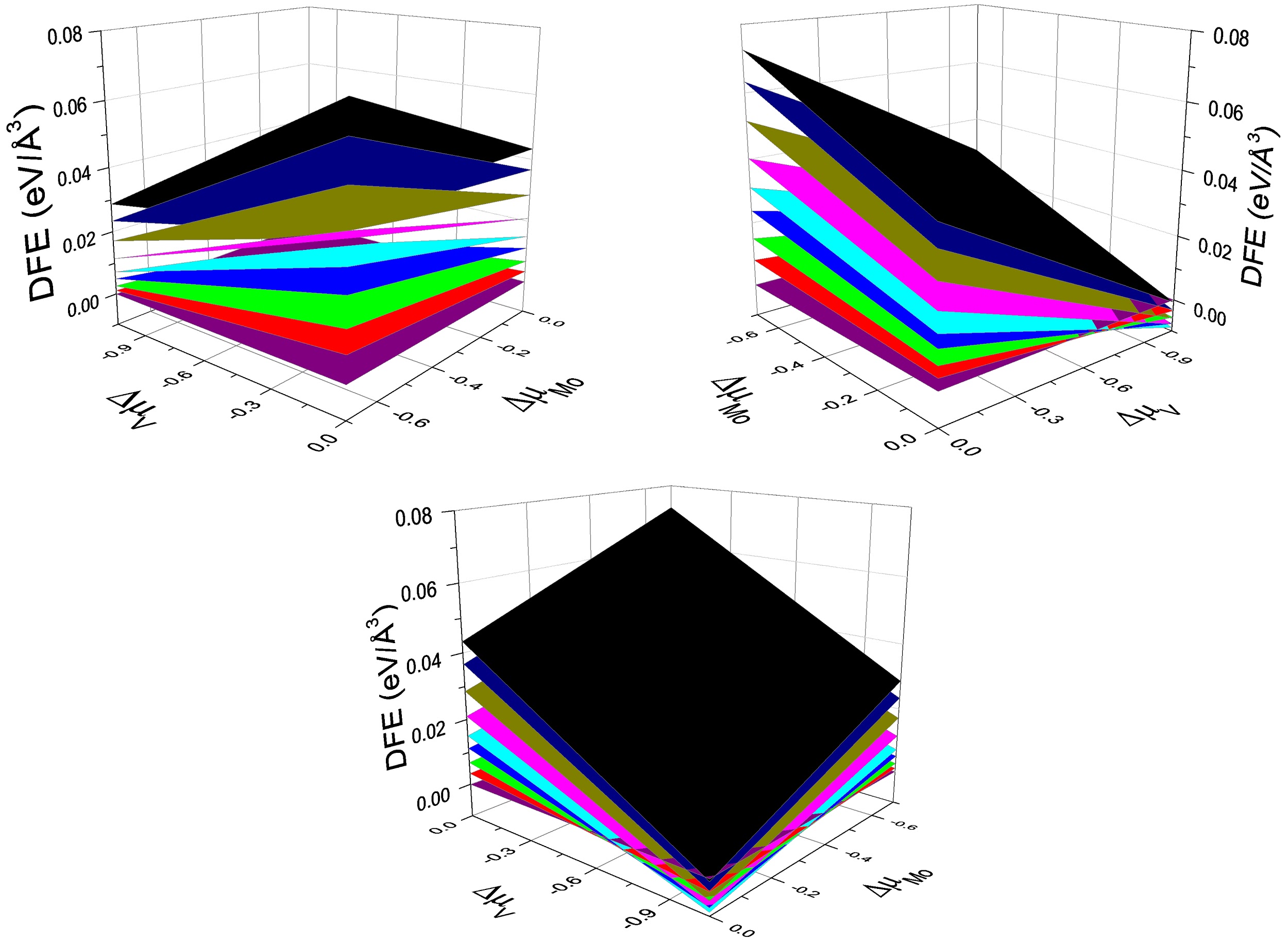
**Figure S4**. DFE graphs in a three-dimensional representation, where each plane represents a different MAX alloy. According to the formalis, the most stable modes have the lowest energy values.

The ELF line profiles corresponding to the double-ordered MAX Mo_2_V_2_AlC_3_ alloy are displayed in **figure S5**. It is noticed a change in the bond nature from mainly ionic to mainly covalent, when Mo substitutes the V_I_ sites. On the other hand, the V_II_-C interaction did not change, it remains ionic.


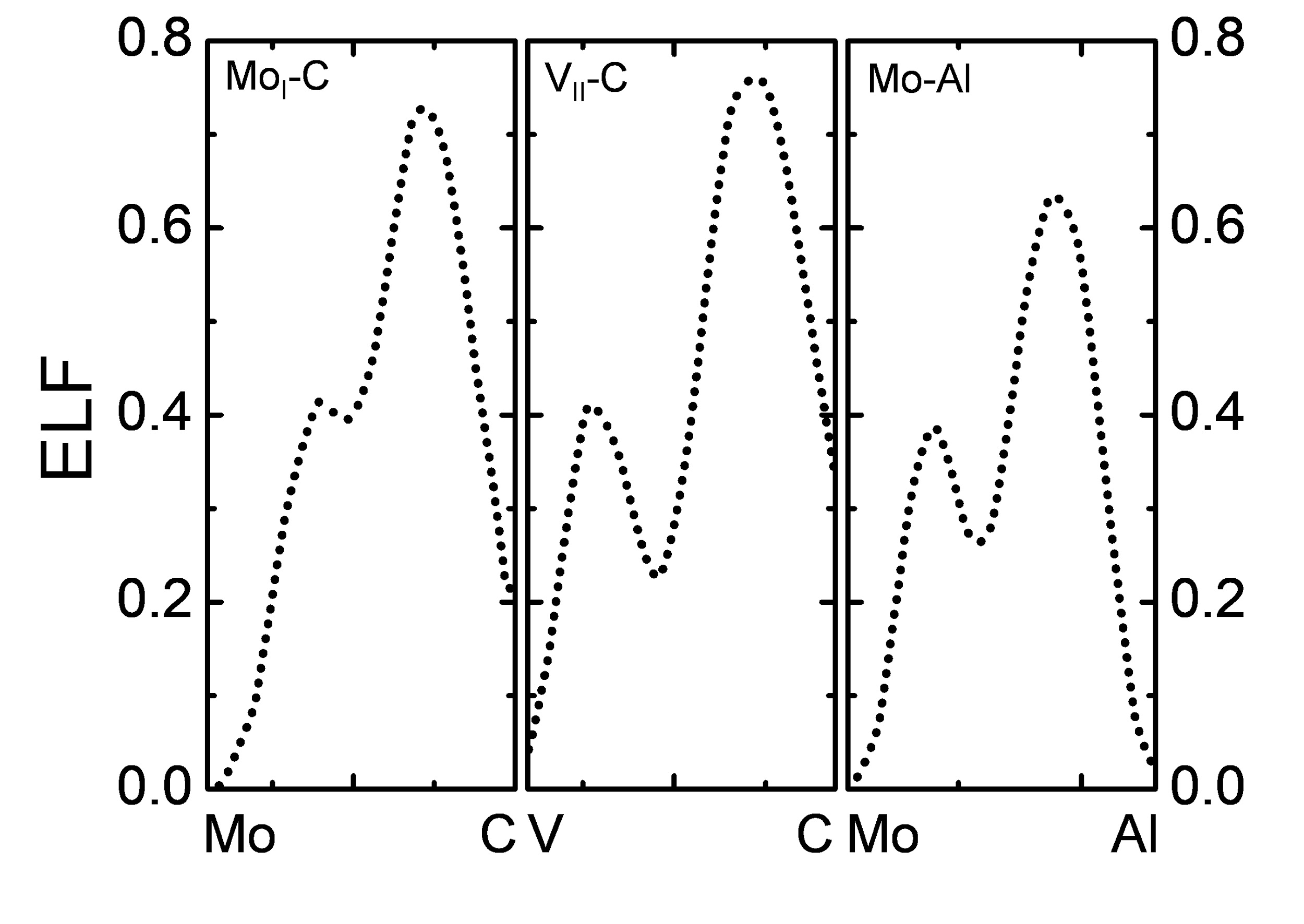


**Figure S5**. ELF line profiles for the interactions present in the double-ordered Mo_2_V_2_AlC_3_ alloy.

The calculated DOS of the Mo_x_V_4-x_AlC_3_ alloys with x= 0.5, 1, 1.5, and 2 are shown in **Figure S6**. The energy reference is the Fermi level. Notice that the main contribution around the Fermi level comes from the V-3d orbitals. Also, the contribution of the Mo-3d orbital increases as Mo content does it.


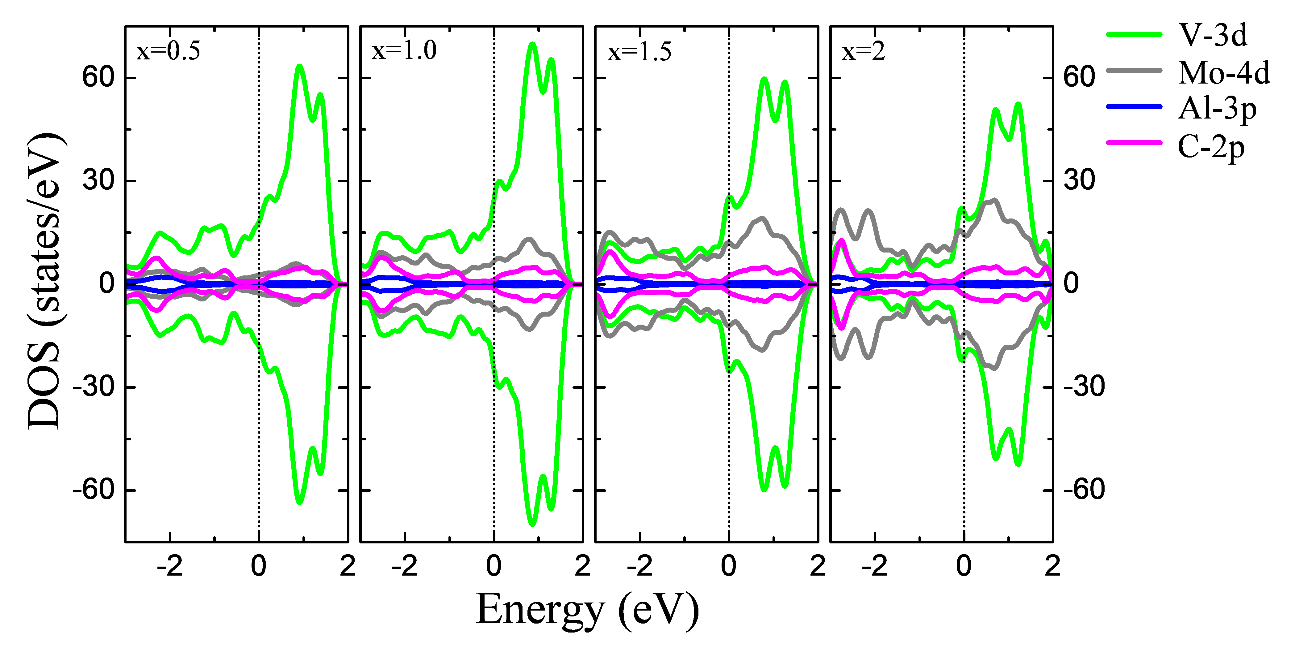


Figure S6. DOS for the most stable structures according to the DFE formalism.
